# Outcome modelling strategies in epidemiology: traditional methods and basic alternatives

**DOI:** 10.1093/ije/dyw040

**Published:** 2016-04-20

**Authors:** Sander Greenland, Rhian Daniel, Neil Pearce

**Affiliations:** 1 Department of Epidemiology and Department of Statistics, University of California, Los Angeles, CA, USA; 2 Department of Medical Statistics, London School of Hygiene and Tropical Medicine, London, UK; 3 Centre for Public Health Research, Massey University, Wellington, New Zealand

## Abstract

Controlling for too many potential confounders can lead to or aggravate problems of data sparsity or multicollinearity, particularly when the number of covariates is large in relation to the study size. As a result, methods to reduce the number of modelled covariates are often deployed. We review several traditional modelling strategies, including stepwise regression and the ‘change-in-estimate’ (CIE) approach to deciding which potential confounders to include in an outcome-regression model for estimating effects of a targeted exposure. We discuss their shortcomings, and then provide some basic alternatives and refinements that do not require special macros or programming. Throughout, we assume the main goal is to derive the most accurate effect estimates obtainable from the data and commercial software. Allowing that most users must stay within standard software packages, this goal can be roughly approximated using basic methods to assess, and thereby minimize, mean squared error (MSE).

**Key Messages**

- The main goal of a statistical analysis of effects should be the production of the most accurate (valid and precise) effect estimates obtainable from the data and available software.
- This goal is quite different from that of variable selection, which is to obtain a model that predicts observed outcomes well with the minimal number of variables; this prediction goal is only indirectly related to the goal of change-in-estimate approaches, which is to obtain a model that controls most or all confounding with a minimal number of variables.
- We illustrate some basic alternative modelling strategies that focus more closely on accurate effect estimation as measured by mean squared error (MSE) and which can be implemented by practitioners with limited programming and consulting resources.

## Introduction

 We have recently reviewed traditional approaches to confounder selection for outcome (risk) and treatment (propensity) models, including significance-testing and ‘change-in-estimate’ (CIE) approaches. ^1^ We argued that the main goal of a statistical analysis of effects should be the production of the most accurate (valid and precise) effect estimates obtainable from the data and available software. Allowing that most users must stay within standard software packages, this goal can be roughly approximated using basic methods to minimize estimated mean squared error (MSE). We here provide an illustrated overview of this approach. 

## Scope, aims and assumptions

 As with our initial review, ^1^ our coverage is not intended for highly skilled practitioners; rather, we target teachers, students and working epidemiologists who would like to do better with data analysis, but who lack resources such as R programming skills or a *bona fide* modelling expert committed to their project. Throughout, we assume that we are applying a conventional risk or rate regression model (e.g. logistic, Cox or Poisson regression) to estimate the effects of an exposure variable X on the distribution of a disease variable Y while controlling for other variables, and that the outcome is uncommon enough so that distinctions among risk, rate and odds ratios can be ignored. The other variables include forced variables, such as age and sex, which we may always want to control, and may also include unforced variables about which we are unsure whether to control. 

 We also assume that data checking, description and summarization have been done carefully. ^2^ Finally, we assume that all quantitative variables have been: re-centreed to ensure that zero is a meaningful reference value present in the data; and rescaled so that their units are meaningful differences within the range of the data; ^3^ and that univariate distributions and background (contextual) information have been used to select categories or an appropriately flexible form (e.g. splines) for detailed modelling. ^3^

 Elsewhere we have discussed the issues involved in simply adjusting for all measured potential confounders. ^1^ This approach can be valid when the number of covariates is not too large in relation to the study size and the included covariates are not highly predictive of exposure. Nonetheless, controlling too many variables can lead to or aggravate problems arising from data sparsity or from high multiple correlation of exposure with the controlled confounders (which we term multicollinearity), in which case one may seek to reduce the number of modelled covariates. 

 There are of course variables for which control may be inappropriate based on preliminary causal considerations. These include intermediates (variables on the causal pathway between exposure and diseases) and their descendants ^4^ and any other variable influenced by the exposure or outcome. ^5–7^ These also include variables that are not part of minimal sufficient adjustment sets, whose control may increase bias. ^4–11^ We assume that these variables have been identified and eliminated e.g. using causal diagrams ^4^^,^^6^^,^^8^ to display contextual theory, ^12^ leaving us with a set of potential adjustment covariates (often called ‘potential confounders’), including those variables that we are reasonably confident would reduce bias if controlled and our study size were unlimited. We focus only on basic selection from these variables, leaving aside many difficult issues about model specification and diagnostics, ^3^^,^^13–19^ time-varying exposures and confounders, interactions and mediation. ^20–23^

## Multicollinearity and mean squared error: modified CIE approaches

One issue that is not explicitly considered or discussed in most epidemiological strategies is that of multicollinearity of covariates with exposure, i.e. when exposure is nearly a linear combination of other variables in the model. This problem becomes most obvious in propensity-score analyses when the exposure is so well predicted that there is little overlap in the exposed and unexposed scores. With multicollinearity, exposure effect estimates become unstable, as reflected by large standard errors.

 To combine bias and variance considerations when dealing with genuine confounders, consider estimation of an exposure effect measure represented by a single coefficient *β* , such as a rate difference or log risk ratio. The bias B in an estimator of *β* is the difference between the expected value (mean) *μ* of the estimator and the ‘true’ population value *β* , so B =  *μ – β* . The standard error (SE) of the estimator is just its standard deviation around that mean *μ* ; SE ^2^ is thus the estimator’s variance. The mean squared error (MSE) of the estimator of *β* combines these properties via the equation MSE = B ^2^ + SE ^2^ . ^24–27^ Reducing multicollinearity by dropping variables can decrease the variance (SE ^2^ ) component of the MSE, but may also increase the bias B in the estimator of *β* if the dropped variables are indeed necessary to adjust for, given the retained variables. Thus we seek ways of reducing the SE of the estimator (e.g. by removing a source of multicollinearity) without seriously increasing its bias B, so that the MSE is reduced. ^24^^,^^25^^,^^27^

 Several formal methods seek to minimize MSE in effect estimation with uncertain confounders, but require special programming. ^19^^,^^28^^,^^29^ We will describe a more crude approach that extends ordinary CIE approaches ^1^ to consider estimated MSE minimization using ordinary software outputs. Suppose we selectively delete confounders from a full model and see what happens to the exposure coefficient estimate and its standard error. Assuming the full-model estimate is unbiased, we can then estimate the bias B _reduced_ from the deletion by the difference between the reduced-model estimate $\hat{\beta }$_reduced_ and full-model estimate $\hat{\beta }$_full_ . This step leads to the following equations for estimating the change in MSE (ΔMSE) from reducing the model by deleting the confounder: 
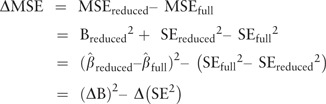

where (ΔB) ^2^ estimates the squared-bias increase from the deletion and Δ(SE ^2^ ) estimates the variance decrease from the deletion. A positive difference, i.e. (ΔB) ^2^ > Δ(SE ^2^ ), indicates that the deletion increased the MSE; a negative difference indicates that the deletion reduced the MSE. We say ‘indicates’ because, of course, we have only rough estimates of B, SE and MSE, and $\hat{\beta }$_full_ , which will be approximately unbiased only when the model, the set of measured confounders and the sample size are all sufficient for approximate validity. This approach is illustrated in Box 1, with an example involving two correlated variables, sodium and potassium intake.

Box 1

We consider an example from a study of sodium intake in infancy (age 4 months) and blood pressure at 7 years. ^30^ The analysis involved adjusting for a relatively large number of potential confounders (see Table 1 ). A potentially important confounder was potassium intake at the same age, which was strongly correlated with sodium intake (r = 0.81). This was reflected in an increase in the standard error for the sodium coefficient when potassium was also included in the model. ^30^ The authors therefore note that ‘due to high sodium-potassium correlations, effect of sodium independent of potassium could not be estimated with reasonable precision’, and they therefore did not control for potassium in the analyses.

We did RMSE analyses ( Table 1 ), which showed that although there was an increase in the SE of the sodium coefficient when potassium is included in the model (compare model 1 with model 2a), the reduction in SE from deleting potassium from the model is offset by the increase in bias (sodium RMSE = 0.294 with potassium excluded vs 0.290 with potassium included). Thus, controlling for potassium appears to be no worse in accuracy, in addition to having smaller approximate bias.

Next, consider potassium as the main exposure: we obtain a lower RMSE (0.095) for the potassium coefficient when including sodium compared with excluding sodium (0.130); thus controlling for sodium appears to be preferable.

**Table 1.** Associations of sodium and potassium intake at age 4 months with blood pressure (BP) at age 7 years ^29^

Model	Exposure variables *	Coefficient estimate	SE for coefficient	Coefficient bias estimate	Indicates bias	Indicates large collinear	Root MSE estimate *
1	Sodium	0.518	0.290	Referent			0.290
	Potassium	0.099	0.095	Referent			0.095
2a	Sodium	0.708	0.225	0.190	Yes	Yes	0.294
2b	Potassium	0.206	0.074	0.107	Yes	Yes	0.130

*All analyses are adjusted for energy intake at 4 or 8 months, age at BP measurement, sex, socioeconomic position (maternal and paternal education), family social class, maternal age at childbirth, parity, birthweight, gestational age, breastfeeding, smoking during pregnancy, sodium intake at 7 years.

 As with CIE, the exposure-coefficient change resulting from covariate deletion can be assessed by examining the estimated change directly, and also with a collapsibility test, i.e. a test of the hypothesis that the deletion does not change the exposure coefficients. ^31–33^ One caution to these approaches is that an accurate assessment of confounding may require examining changes from moving groups of variables. Regardless of the number of covariates being deleted, however, if there is one exposure term X, then a one degree of freedom chi-squared statistic for this hypothesis is χ _c_^2^  = (ΔB) ^2^ /Δ(SE ^2^ ). ^33^ Deleting a variable when ΔMSE > 0 is equivalent to deleting the variable when χ _c_^2^ < 1, which corresponds to *P* > 0.32 for collapsibility. Appendix 1 (available as Supplementary data at *IJE* online) gives further details, describes a generalization of this test to exposures represented by multiple terms and suggests avenues for improvement. 

 To illustrate the general algorithms, denote by W _1_ ,…,W _J_ those variables (such as age and sex) that we want forced into all our models along with exposure X because they are expected to be important confounders or modifiers of the exposure effect measure, or because they are known strong risk factors that everyone wants to see in adjustment; this list could include age splines, sex and ethnicity indicators etc. Our chief concern will be with the remaining variables U _1_ ,…,U _H_ , whose importance for adjustment is highly uncertain. 

 Some hypothetical modelling results are shown in Table 2 . We suppose result 1 is from a full model for the disease rate with exposure, the forced variables and all potential confounders. Results 2a–d then illustrate the four mutually exclusive possible outcomes of comparing a full (maximal) model including the potential confounders (forced and unforced variables) with a minimal model including only the main exposure and the forced variables. Result 2a suggests little or no confounding or multicollinearity problems, since there is little difference between the basic and full models; we might therefore prefer the simplicity of reporting estimates from the minimal model. In contrast, result 2b suggests there is confounding by the unforced variables, as seen by contrasting the exposure rate ratios from model 1 and model 2b, indicating that it is necessary to control at least some of the unforced variables. 

**Table 2.** Hypothetical results from rate regressions in which a covariate is or is not a confounder or a source of multicollinearity

Model	Model variables	Exposure coefficient estimate	Rate ratio estimate	SE for coeff.	95% CL	Coefficient bias estimate *	Indicates bias?	Indicates strongly collinear?	Root MSE estimate *	Collapsibility χ ^2^ and *P* -value ^33^
1	X,W _1_ …W _J_ , U _1_ …U _H_	0.693	2.00	0.24	1.25,3.20	Referent			0.24	
** Some mutually exclusive alternative possibilities under model 2 (minimal model in which all unforced variables U _1_ …U _H_ are dropped) **										
2a	X,W _1_ …W _J_	0.693	2.00	0.24	1.25, 3.20	0	No	No	0.24	0, *P* = 1
2b	X,W _1_ …W _J_	1.099	3.00	0.20	2.03, 4.44	0.405	Yes	No	0.45	9.34, *P* = 0.002
2c	X,W _1_ …W _J_	0.693	2.00	0.14	1.52, 2.63	0	No	Yes	0.14	0, *P* = 1
2d	X,W _1_ …W _J_	1.099	3.00	0.14	2.28, 3.95	0.405	Yes	Yes	0.43	4.03, *P* = 0.04

*Taking model 1 as the referent (‘gold standard’).

Results 2c and 2d involve large multicollinearity, as indicated by the difference (0.14 compared with 0.24) in the standard error for the main exposure coefficient. The more favourable situation is when the factors causing multicollinearity are very weak confounders, so they can be deleted from the model without increasing the MSE of the exposure-effect estimate. This situation is indicated when deleting these factors leaves the exposure-effect estimate virtually unchanged, but greatly reduces its standard error (as in result 2c), suggesting that the minimal model provides more accurate estimates of the exposure effect (i.e. it has a smaller MSE). Again, we caution that this smaller standard error does not account for the preliminary testing and is thus too small by an unknown amount.

 It is more difficult to proceed when multicollinearity arises from a strong confounder (result 2d), since the increase in precision due to deleting such a confounder may be more than offset by an increase in confounding. ^26^ We thus must consider the net impact of reducing the SE of the exposure-effect estimate while increasing its bias, and we do so by directly comparing square roots of estimated MSE (RMSE); we use the square roots to put the results back on the scale of the effects and biases. 

 In result 2d, the estimated RMSE from the minimal model is substantially larger (0.43) than from the full model (0.24), because the minimal model involves a large increase in confounding and a relatively smaller decrease in multicollinearity. The task is then to identify a compromise model (including some but not all the variables in question) in which multicollinearity is reduced, but there is negligible increase in confounding. This could occur, for example, if the variables most responsible for confounding were distinct from the variables most responsible for multicollinearity. Candidate variables can be assessed by dropping each variable in turn from the full model. Of course, this process may fail to identify any acceptable model reduction, in which case the options are to stay with the full model or else turn to more sophisticated methods such as penalized estimation or hierarchical (multilevel or mixed) models to improve accuracy. ^13^^,^^34–37^

Table 1 gives effect estimates without and with adjustment for the U _h_ , which provides a basis for discussing the plausibility of residual confounding. For example, if adjustment using imperfectly measured U _h_ removes more than one-half of the excess rate associated with a particular main exposure, then it is reasonable to speculate that adjustment with better U _h_ information would have removed most of the excess rate. Thus it can be worthwhile to present estimates from different degrees of adjustment. 

Based on the above considerations, Box 2 outlines one backward-deletion strategy for screening out potential confounders. This strategy is intended as a set of options, rather than a prescription; it would be applicable in settings in which a full model can be fit without problems, there is not an inordinate number of potential confounders to consider and there is no clear and strong heterogeneity. One implementation is as follows:

B1) Fit the full model, with no exposure-covariate products. This model provides an average regression across the included covariates, even if heterogeneity is present. ^38–40^
B2) Enter the following reduction loop, starting with the full model as the ‘current model’:
  - For each candidate variable that remains in the current model, re-run the model without its terms (the U _h_ that represent it) and compute the resulting ΔMSE relative to the current model from dropping those terms; again,
    $$\Delta \text{MSE }=\;{\left({\hat{\beta }}_{\text{reduced}}–{\hat{\beta }}_{\text{current}}\right)}^{\text{2}}–\;\left({\text{SE}}_{\text{current}}{}^{\text{2}}–{\text{ SE}}_{\text{reduced}}{}^{\text{2}}\right)$$
  - If any candidate in the model has ΔMSE < 0 (indicating its deletion reduces MSE), drop the one with the smallest (most negative) ΔMSE and go to step (a) if there is any candidate left in the model. Otherwise (if there is no candidate U _h_ left in the model, or none left have ΔMSE < 0), stop and use the current model.

Box 2 Variable selection based on backward deletion using estimated MSE reduction

1.   **Baseline specification**
  1.1 Select the variables that are appropriate to include, using a causal directed acyclic graph (DAG) to exhibit theorized causal relations among variables identified *a priori* as potentially important for estimating the effects of interest.
  1.2 Divide the variables into three classes: (i) the main exposure X; (ii) forced-in variables (e.g. age, sex) which are always included in the model (W _1_ …W _J_ ); and (iii) the non-forced variables which will be candidates for deletion (U _1_ …U _H_ ).
  1.3 Run a ‘full’ model including all main exposure terms, forced-in variables and non-forced variables from 1.3, with no exposure-covariate products. [If full model does not converge or the results indicate sparse-data bias, change to a forward-selection strategy, or use hierarchical (multilevel or mixed) or penalized modelling methods.]
2.   **Variable selection** Enter the following reduction loop, starting with the full model as the ‘current model’:
  2.1 For each candidate variable that remains in the current model, re-run the model without its terms (the U _h_ that represent it)and compute the resulting ΔMSE relative to the current model from dropping those terms:
    $${\left({\hat{\beta }}_{\text{reduced}}–{\hat{\beta }}_{\text{current}}\right)}^{\text{2}}–\;\left({\text{SE}}_{\text{current}}{}^{\text{2}}–{\text{ SE}}_{\text{reduced}}{}^{\text{2}}\right)$$
  2.2 If any candidate has ΔMSE < 0, drop the one with the smallest (most negative) ΔMSE and go to step 4.2 if there are any candidates left in the model. Otherwise (if there is no candidate U _h_ left in the model, or none left have ΔMSE < 0), stop and use the current model.
3.   **Assessment of heterogeneity (effect-measure modification)**
  3.1 Assess heterogeneity in a series of supplementary analyses, focusing on covariates of a priori interest

We can also derive a parallel forward-selection strategy starting with the basic model when there are more potential confounders to consider than can reasonably fit at once (e.g. when using too many of them results in sparse-data bias, thus spuriously inflating (ΔB) ^2^ ):

F1) Fit the basic model, with no exposure-covariate products.
F2) Enter the following expansion loop, starting with the basic model as the ‘current model’:
  - For each candidate variable that is not in the current model, re-run the model expanded with its terms U _h_ and compute the ΔMSE from adding those terms.
  - If any candidate U _h_ not in the model has ΔMSE > 0 (indicating its addition reduces MSE), enter the one with the largest ΔMSE and go to step (a) if any candidate remains left out. Otherwise (if there are no more unselected candidates, or if none left out have ΔMSE > 0), stop and use the current model.

Both the above approaches can be viewed as a modification of conventional testing strategies in one major way: the test of the confounder coefficient is replaced by a test of collapsibility of the exposure coefficient over the confounder. This test is easily constructed from ordinary outputs (see Appendix 1, available as Supplementary data at *IJE* online) and is appropriately sensitive to the confounder relation to exposure as well as to its relation to disease. It can also be viewed as a modification of CIE strategy that allows for random error in the observed change and for the possible variance reduction from deletion. 

In Box 3, these approaches are applied to a study of atopy in Poland, and their results are compared with other common approaches.

Box 3

We consider an example from a study of the prevalence of atopy in a small town and neighbouring villages in Poland in 2003. ^41^ In the current analysis, we estimate the association between ‘no current unpasteurized milk consumption’ and current atopy status. It was plausible that lack of unpasteurized milk consumption could increase the risk of atopy. Because drinking unpasteurized milk happens mostly in rural settings, however, there are a number of other exposures which may be related to both unpasteurized milk consumption and the prevalence of atopy.

Main exposure: never drinking unpasteurized milk (1: never vs 0: regularly/sometimes).

Forced variables: age-group (seven categories), sex.

Potential confounders:

Live in town (yes/no) or village
Live on a farm (yes/no)
Contact (regular/occasional) with cows, pigs, poultry, sheep or goats, horses
Work (regular/occasional) milking cows, cleaning barns, collecting eggs
Firstborn (yes/no)
Number of siblings (1, 2, 3+)
Current smoker (yes/no)
Lived in town (yes/no) or village as a child
Lived on a farm (yes/no) as a child
Parents were farmers (yes/no)
Family kept cows, pigs, poultry, sheep or goats, horses.

*Basic model*

Model 1 in Table 3 shows the results of the basic analysis for milk, adjusted for the forced variables (age-group and sex).

*Full model*

Model 2a in Table 3 shows the results of the full maximum likelihood (ML) model, adjusting for all potential confounders; there is a substantial change in the odds ratio for milk (from 2.46 to 1.50), but there is also an increase in the SE for the coefficient estimate (from 0.225 to 0.257). Model 2b is the full model fit using the Firth adjustment for coefficient-estimate bias. ^42^^,^^43^ This is used as the ‘standard’ to estimate the bias of the other models, and is combined with the bootstrap SEs to estimate the RMSE. Overall, the milk coefficients from the full models have a much lower RMSE (0.262, 0.251) than in the basic model (0.567) because the increase in SE from including all potential confounders is small in comparison with the change in the coefficient estimate.

*Traditional stepwise regression*

Model 3a in Table 1 shows the results of a forwards stepwise logistic regression (using *P* < 0.20 as the criterion for inclusion) with milk, age group and sex as forced variables; Town, Firstborn, Current smoker, Town as a child, Parents farmers, Parents kept poultry and Parents kept horses were also selected. Model 3b is again a forwards stepwise logistic regression but uses *P* < 0.05 as the criterion for inclusion. Model 3c and d are the backwards stepwise procedures with *P* < 0.20 and *P* < 0.05, respectively.

*AIC*

Model 4a in Table 1 shows the results of using the Akaike Information Criterion (AIC) ^14^ where variables were forward selected to achieve the largest increase in AIC at each step. Model 4b is from using AIC for backwards deletion.

*BIC*

Model 5a and b was selected in parallel to 4a and b but using the Bayesian Information Criterion. ^14^

*Relative change-in-estimate approach*

Only town residence (in addition to the forced variables of age group and sex) produced a substantial change in the estimate for milk; once this was in the model, no other variable changed the milk odds ratio estimate by more than 10%, leading to model 6a. Model 6b is from the analogous backwards procedure and resulted in the same model.

*RMSE*

Model 7a in Table 1 shows the results of using RMSE reduction for forward selection in two different ways. Model 7a1 used (at each step) the larger of the two models being compared as the reference for estimating RMSE reduction, and is thus analogous to the other procedures, whereas model 7a2 used the full model as the reference for each step. Model 7b is the backwards version of the same procedure. Model 7b1 used (at each step) the larger of the two models being compared as the reference (for estimating the RMSE), whereas model 7b2 used the full model as the reference for each step.

*Penali* *z* *ation*

Following previous recommendations, ^37^^,^^44^ we included two analyses with weakly informative shrinkage priors for each coefficient. The first analysis used a log-F(1,1) (Haldane) prior distribution for each coefficient, which is equivalent to using an F(1,1) prior distribution for the odds ratio (antilog) from each coefficient, and assigns 95% probability to the odds ratio falling between 1/648 and 648. The second analysis used a log-F(2,2) (standard logistic) distribution for each coefficient, which is equivalent to using an F(2,2) prior distribution for the odds ratio from each coefficient, and assigns 95% probability to the odds ratio falling between 1/39 and 39. The priors were imposed by adding two pseudo-observations for each coefficient to the actual data file, with weights of ½ for the F(1,1) prior and weights of 1 for the F(2,2) prior, then fitting the full model to the augmented data set by maximum likelihood, with the constant term replaced by an indicator for ‘actual-data record’ and weights of 1 for all actual-data records. ^36^^,^^45^^,^^46^

*Discussion*

In this example, all of the modelling approaches yielded reasonably similar findings—the full model (Firth bias-adjusted) yielded an OR of 1.47, and all of the other approaches produced ORs in the range of 1.42 to 1.51. The RMSEs were also similar, smaller than that of the full model and substantially smaller than that for the basic model. The fact that there exist models with lower estimated RMSE than the models selected by the RMSE procedures 7ab (using the larger of the two models as the reference) illustrates how a procedure that selects or rejects variables one at a time (forwards or backwards) does not always find the model with the overall optimal value of the criterion being used.

In this example, Town is the only variable whose inclusion/exclusion in the model has much impact on the exposure effect estimate. Town is also highly predictive of the outcome. Thus, all methods select it, and whatever else they happen to select makes very little difference for any of the measures considered. For the same reasons, the bootstrap 95% CIs (which take variable selection into account) were in general only slightly larger than the ‘standard’ 95% CIs. We therefore see little apparent advantage of one method over another in this example. Nonetheless, in a setting with strong confounding by intercorrelated groups of multiple confounders, we might find more stark differences among the results from different methods.

**Table 3.** Model-adjusted associations of current unpasteurized milk consumption with current atopy status ^40^

Model	Model variables *	Exposure coefficient estimate	SE for coefficient	OR	95% CL for OR	Estimated bias and RMSE	Bootstrap SE ^†^ for coefficient	Bootstrap 95% CL ^‡^ for OR
1 (basic)	Milk	0.899	0.225	2.46	1.58, 3.82	0.516 0.567	0.236	1.59, 3.97
2a (ML full)	Milk All other variables ^#^	0.406	0.257	1.50	0.91, 2.48	0.023 0.262	0.261	0.89, 2.46
2b (Firth) ^42^^,^^43^	Milk All other variables ^#^	0.383	0.252	1.47	0.91, 2.40	0.000 0.251	0.251	0.89, 2.37
3a (forwards stepwise, *P* < 0.20)	Milk Town Firstborn Current smoker Town as a child Parents farmers Parents kept poultry Parents kept horses	0.390	0.244	1.48	0.91, 2.38	0.007, 0.261	0.261	0.87, 2.43
3b (forwards stepwise, *P* < 0.05)	Milk Town Current smoker Town as a child Parents kept poultry	0.383	0.243	1.47	0.91, 2.36	<0.001 0.261	0.261	0.88, 2.44
3c (backward stepwise, *P* < 0.20)	Milk Town Firstborn Current smoker Parents farmers Parents kept poultry Parents kept horses	0.398	0.244	1.49	0.92, 2.40	0.015 0.261	0.261	0.88, 2.47
3d (backward stepwise, *P* < 0.05)	Milk Town Current smoker Parents farmers Parents kept poultry Parents kept horses	0.414	0.244	1.51	0.94, 2.44	0.031 0.265	0.263	0.93, 2.61
4a (forwards AIC)	Milk Town Horses Firstborn Current smoker Parents kept poultry	0.381	0.243	1.46	0.91, 2.36	−0.002 0.260	0.260	0.86, 2.39
4b (backward AIC)	Milk Town Horses Firstborn Current smoker Parents kept poultry Parents kept horses Parents farmers	0.398	0.244	1.49	0.92, 2.40	0.015 0.262	0.262	0.88, 2.48
5a (forwards BIC)	Milk Town Current smoker Parents kept poultry	0.393	0.243	1.48	0.92, 2.39	0.010 0.264	0.264	0.88, 2.45
5b (backward BIC)	Milk Town Current smoker Parents kept poultry	0.393	0.243	1.48	0.92, 2.39	0.010 0.264	0.264	0.87, 2.45
6a (forwards CIE)	Milk Town	0.400	0.242	1.49	0.93, 2.39	0.017 0.255	0.254	0.93, 2.56
6b (backward CIE)	Milk Town	0.400	0.242	1.49	0.93, 2.39	0.017 0.255	0.254	0.92, 2.52
7a (forwards RMSE, larger model as referent)	Milk Town Poultry Collecting eggs Number of siblings Parents kept cows Parents kept poultry	0.363	0.245	1.44	0.89, 2.32	−0.020 0.258	0.257	0.86, 2.35
7b (backward RMSE, larger model as referent)	Milk Town Poultry Collecting eggs Firstborn	0.350	0.243	1.42	0.88, 2.29	0.017 0.257	0.256	0.84, 2.28
8a (forwards RMSE, full model as referent)	Milk Town	0.400	0.242	1.49	0.93, 2.39	−0.033 0.263	0.261	0.88, 2.45
8b (backward RMSE, full model as referent)	Milk Town Parents kept cows Parents kept poultry	0.407	0.242	1.50	0.94, 2.42	0.024 0.264	0.263	0.89, 2.51
9a penalization by log-F(1,1) priors ^§^^45^	Milk All other variables ^#^	0.396	0.253	1.49	0.90, 2.44	0.013 0.253	0.253	0.90, 2.42
9b penalization by log-F(2,2) priors ^¶^^45^	Milk All other variables ^#^	0.389	0.250	1.47	0.90, 2.41	0.006 0.246	0.246	0.90, 2.36

*All analyses are adjusted for age group and sex.

^†^ Based on 4000 bootstrap samples.

^‡^ Bias-corrected and accelerated (BCa) with 4000 resamples. ^56^

^#^ Town, farm, cows, pigs, poultry, sheep/goats, horses, milking cows, cleaning barns, collecting eggs, firstborn, number of siblings, current smoker, lived in town or village as a child, parents were farmers, family kept cows, family kept pigs, family kept poultry, family kept sheep or goats, family kept horses.

^§^ Equivalent to F(1,1) prior for odds ratio; 95% prior limits are 1/648, 648.

^¶^ Equivalent to F(2,2) prior for odds ratio; 95% prior limits are 1/39, 39.

## Some limitations

 As with most variable-selection procedures including stepwise and CIE, confidence intervals obtained by combining the final point estimate and SE from the above strategy are not theoretically valid. Simulation studies ^24^^,^^25^ so far suggest that this invalidity is negligible in typical settings, due to the high significance level and therefore liberal inclusion implicit in using ΔMSE = 0 as the decision point. Nonetheless, the strategy could be improved by using bootstrapping or cross-validation to estimate ΔMSE and set confidence intervals. 

 A further problem with using CIE strategies for logistic regression is that it is possible the change in estimate is largely due to more sparse-data bias (i.e. too few subjects at crucial combinations of variables) in the full-model estimate $\hat{\beta }$_full_ rather than increased confounding in the reduced-model estimate $\hat{\beta }$_reduced_ . For a binary exposure X and disease Y, this problem becomes noticeable when there are much fewer than about 4 subjects per confounder coefficient at each exposure-disease combination; for example, with 7 confounder terms we would want at least 4(7) = 28 subjects in each cell of the two-way XY table for some assurance that sparse-data bias in $\hat{\beta }$_full_ is small. One way to avoid this problem is to switch to penalized estimation; it is also possible to apply the above reduction algorithms after minimal penalization to reduce sparse-data bias. ^44–48^

 Another problem however is that logistic coefficients are in general not collapsible, in that there will be differences between the actual (underlying) coefficients with and without a given covariate if the covariate predicts the outcome, even if that covariate is not a confounder by virtue of being independent of exposure. ^6^ This difference will be negligible unless the outcome is common, in which case it will be advisable to switch to estimation of collapsible effect measures (such as risk ratios and differences), e.g. by regression standardization. ^13^

## Discussion

 Like more sophisticated but computationally intensive methods, ^19^ the strategies we describe differ from stepwise regression and other purely predictive approaches, in that their goal is to improve accuracy of exposure effect estimates rather than to simply predict outcomes. At the same time, recognizing that the gap between state-of-the-art methods and what is done in most publications has only grown over time, they are intended to fall within the scope of the limits on software and effort that constrain typical researchers. Thus, parsimony is replaced by the goal of minimizing error in effect estimation. 

 A related point is that, as with parsimony, pursuit of goodness-of-fit may lead to inappropriate decisions about confounder control; in particular, some variables may not be included in the model because they do not significantly improve the fit, even though they are important confounders. ‘Global’ tests of fit are especially inadequate for confounder selection ^13^ since there can be many ‘good-fitting’ models that correspond to very different confounder effects and exposure effect estimates. ^26^

 Parsimony and goodness-of-fit are helpful only to the extent they reduce variance and bias of the targeted effect estimate. The general inappropriateness of parsimony as a goal in causal analysis is supported by simulation studies in which full-model analysis has often outperformed conventional selection strategies. ^24^^,^^25^^,^^27^ This result raises the question: if we can control for all potential confounders, then why wouldn’t we? If indeed we have numbers so large that there is no problem from controlling too many variables, we would generally expect covariate elimination to provide little benefit for the accuracy of effect estimates. But the harsh reality is that even databases of studies with hundreds of thousands of patients often face severe limits in crucial categories, such as the number of exposed cases. Coupled with the availability of what may be hundreds or even thousands of variables, some kind of algorithmic approach to potential confounders becomes essential. ^49^^,^^50^ The strategies we describe are designed for common borderline situations in which control of all the variables may be possible, but some accuracy improvement may be expected from eliminating some or all variables whose inclusion is of uncertain benefit. 

 A number of criticisms can be made of the MSE-based strategy in Box 2. First, it can be argued that any data-based model reduction will produce biased estimates because it depends on the assumption that it is not necessary to control the omitted variables (conditional on control of the included variables). ^51^ We regard this criticism as somewhat misguided insofar as every epidemiological estimate suffers from some degree of bias from uncontrolled confounders, differential subject selection and measurement error (in both exposures and confounders); the key question is then whether the bias from omitting a variable is of contextual importance. 

 Second, as we have emphasized, simple selection methods (such as stepwise, CIE and apparent MSE change) do not take account of random variability introduced by data-based model selection. Thus, without cross-validation or some other adjustment, the standard error of the resulting effect estimate is not correctly estimated by taking the standard error computed from the final model. ^15^ With methods that focus on the effect estimate, however, the eliminated variables are generally those that have only weak relations to exposure or disease, the resulting problem is limited. ^25^ Where such problems are of concern, they can be mitigated by the use of shrinkage, penalization and related hierarchical methods, ^13^^,^^14^^,^^34–36^^,^^45^^,^^46^^,^^52^^,^^53^ model averaging, ^54^^,^^55^ cross-validation ^19^ or bootstrapping. ^56^

 Third, the MSE approaches we describe may encounter technical difficulties in precisely the situation of most concern here, namely when there is multicollinearity. As we mentioned, sparse-data bias is a chief concern along with related artefacts due to sample-size limitations, which again suggests using in the MSE algorithms the bias-reduced estimates available in commercial software. ^45^^,^^46^

The strategies we have presented in this paper are in no sense optimal; rather they are rough but transparent heuristics which attempt to mitigate some of the difficulties of common approaches without introducing too much new machinery or subtle statistical concepts. Regardless of the strategy adopted, however, it is important that authors document how they chose their models, so that readers can interpret their results in light of the strengths and weaknesses attendant on the strategy that they used.

## Funding

R.D. acknowledges support from a Sir Henry Dale Fellowship jointly funded by the Wellcome Trust and the Royal Society (Grant Number 107617/Z/15/Z). The Centre for Public Health Research is supported by a Programme Grant from the Health Research Council of New Zealand. The research leading to these results has received funding from the European Research Council under the European Union’s Seventh Framework Programme (FP7/2007-2013) / ERC grant agreement no 668954.

## Supplementary Material

Supplementary Data
