# Supplementary material for: Outcome modelling strategies in epidemiology: traditional methods and basic alternatives
Source: Int J Epidemiol. 2016 Apr 20;45(2):565–75. doi: 10.1093/ije/dyw040 (PMC4864881; doi:10.1093/ije/dyw040)
Supplement: Supplementary Data [file dyw040_suppl_data.pdf]

Appendix 1. Relation of CIE and ΔMSE to Collapsibility Tests

Assuming that the target parameter is the exposure coefficient β_{full}, the change in the exposure-effect estimate upon deletion of a variable, $\hat{\beta}_{\text{reduced}} - \hat{\beta}_{\text{full}}$, yields a test statistic for the hypothesis the deletion produced no bias (true $B_{\text{reduced}} = 0$), which is just the collapsibility-test statistic[31-33]

$$\chi_c^2 = (\hat{\beta}_{\text{reduced}} - \hat{\beta}_{\text{full}})^2/(SE_{\text{full}}^2 - SE_{\text{reduced}}^2) = (\Delta B)^2/\Delta(SE^2).$$

This statistic appears unusual since it has a variance difference in the denominator. Nonetheless, if the confounders being tested have an association with the disease under the full model, χ_c^2 has an approximate 1 degree of freedom chi-squared distribution under the hypothesis that their elimination makes no difference for the exposure-disease association. If the denominator is not positive, that indicates no variance reduction from deletion and so we set $\chi_c^2 = 0$.[33] This simple test can be used in place of ordinary testing of the confounder coefficient in selection algorithms, in order to take account of the exposure-confounder association as well as the confounder-disease association.[24 25]

Note that $\chi_c^2 > 1$ exactly when dropping the variable appears harmful, i.e.,when $\Delta MSE > 0$. Interestingly, the probability of $\chi_c^2 > 1$ is 0.317 under the null hypothesis of no bias from deletion, which suggests that a variable should be dropped if and only if $P > 0.317$ from this test. This result parallels simulations reporting improvement in performance from using very high α-levels (e.g., 0.20, as opposed to the traditional 0.05) in tests to select or retain potential confounders.[24 25] Nonetheless, here the test is on the change in the exposure coefficient rather than on the size of the confounder coefficient, so it is more directly relevant to and more powerful for the confounding question.

With multiple exposures or multiple exposure terms $X_1,\ldots,X_I$, $\boldsymbol{\beta}$ the vector of their coefficients, and Cov the estimated covariance matrix of the coefficient estimator $\widehat{\boldsymbol{\beta}}$, the above test generalizes to an I-degree-of-freedom statistic

$$\chi_c^2 = (\widehat{\boldsymbol{\beta}}_{\text{reduced}} - \widehat{\boldsymbol{\beta}}_{\text{full}})'(\Delta C)^{-1}(\widehat{\boldsymbol{\beta}}_{\text{reduced}} - \widehat{\boldsymbol{\beta}}_{\text{full}})$$

to evaluate the change in all I coefficients,[32] where $\Delta C = \text{Cov}_{\text{full}} - \text{Cov}_{\text{reduced}}$. The confounder is then eliminated if $\chi_c^2 < I$ or ΔC is singular, or selected if $\chi_c^2 > I$. Other extensions to allow for potential overfitting and differences in higher-order bias or MSE may be worth investigating; for example, the Firth bias-adjusted estimators [42 43]available in SAS [65], could be used for constructing the test statistic.
